# Concerning the article “False-positive Serum Antiglomerular Basement Membrane Antibody due to Bovine Serum Albumin-containing Surgical Adhesive: A Case Report” by Yoshida et al

**DOI:** 10.1016/j.xkme.2025.100985

**Published:** 2025-02-21

**Authors:** Giulia Zorzi

**Affiliations:** Department of Laboratory Medicine, Cliniques Universitaires Saint-Luc, UCLouvain, Brussels, Belgium

To the Editor:

I am grateful to the authors of this article who helped us unravel a somewhat complicated case: a 72-year-old patient presented to the emergency room at the Cliniques Universitaires Saint-Luc, Bruxelles, with a picture of hemoptysis, which justified the request for anti-glomerular basement membrane antibodies; these were tested in our Laboratory with an enzyme-linked immunoassay technique and were strongly positive in two different samples (345 AU and 394 AU). Surprisingly, anti-MPO and anti-PR3 antibodies were also positive, although at very modest levels. In light of these results, we decided to perform the test with a different method (immunodot), and the test results were completely negative. During a discussion with the clinician, these results were then strongly questioned as the patient was diagnosed with an aortopleural fistula of mechanical origin. We know that false positives are documented because of reactivity to bovine serum albumin used in enzyme-linked immunosorbent assay^1^ methods, but how to be sure to not miss something serious? While studying the literature, we found this clinical case that pushed us to analyze the patient’s file to finally discover that a year earlier he had undergone surgery during which Bioglue was used, as in this article.^2^ Although reports of high sensitivity and specificity with enzyme-linked immunosorbent assaytechniques,^3^^,^^4^ these cases warn us about the interpretation of certain results and push us to confirm systematically every positive sample with a free-bovine serum albumin technique. Finally, for a correct diagnosis of anti-glomerular basement membrane disease, there should be a broader and multidisciplinary vision and a strong collaboration between clinicians and the laboratory.
